# Issues, challenges, and the way forward in conducting clinical trials among neonates: investigators’ perspective

**DOI:** 10.1038/s41372-019-0469-8

**Published:** 2019-09-04

**Authors:** Sindhu Sivanandan, Kajal Jain, Nishad Plakkal, Monika Bahl, Tanushree Sahoo, Shirshendu Mukherjee, Yogendra Kumar Gupta, Ramesh Agarwal

**Affiliations:** 1 Jawaharlal Institute of Postgraduate Medical Education and Research (JIPMER), Puducherry, India; 2 All India Institute of Medical Sciences (AIIMS), New Delhi, India; 3 Clinical Development Services Agency, Faridabad, India; 4 Grand Challenges India, Biotechnology Industry Research Assistance Council, New Delhi, India; 5 Translational Health Sciences and Technology Institute, Faridabad, India

## Abstract

Clinical trials are essential to test the safety and efficacy of new treatments in any population. The paucity of drug trials especially in the neonatal population has led to the widespread use of unlicensed or off-label medications, exposing them to the risks of drug toxicity and ineffective treatment. Ethical and operational challenges are no longer considered valid excuses for not conducting drug trials in neonates. We recently participated in a combined phase-2 and phase-3 trial investigating a new indigenous goat lung surfactant extract (GLSE) for the treatment of respiratory distress syndrome (RDS) in preterm neonates. In this article, we share pertinent challenges faced by us during the trial to better inform and foster-positive discussion among drug developers, administrators, regulatory authorities, patient advocacy groups, and researchers. Also, we provide many tools developed for the GLSE trial that can be modified and used by prospective trialists.

## Introduction

Well-designed randomized controlled trials (RCTs) are the gold standard for testing the efficacy and safety of any new intervention. The majority of participants enrolled in trials are adult males with under-representation of women, children, elderly, and ethnic minorities [1]. While the situation seems to be improving with greater representation of women [2], yet fewer clinical trials are conducted among children and neonates. In the absence of child-specific testing of a drug, clinicians tend to extrapolate information from adult studies. The result is unlicensed and off-label prescription of drugs in neonates [3]. Examples include the use of aminophylline for treatment of apnea of prematurity (off-label indication), fentanyl for pain relief (off-label age and dose), and sildenafil for the treatment of pulmonary hypertension in neonates (unlicensed) [4].

## Drug trials in neonates

There is a growing necessity to conduct all stages of drug development trials in neonates [5], as many disease conditions are specific to this population and drugs have variable pharmacokinetic/pharmacodynamic (PK/PD) and safety profiles [6]. Despite the need, drug trials in neonates are difficult to perform. These include ethical challenges like the inherent vulnerability of the neonatal population, their higher risk of mortality and morbidity, and the difficulties in obtaining informed parental consent. The immaturity of organ system affects drug pharmacokinetics in many ways: drug absorption, volume of distribution due to difference in body water composition and fat stores, serum concentrations due to lower levels of binding protein, half-life due to immature metabolic pathways or elimination mechanisms, and neurotoxicity due to the immature blood brain barrier. Other limitations include the difficulty in obtaining blood samples for drug testing and other investigations in a neonate, and the lack of adequate funding [7]. Poor financial incentives from neonatal drug market and the greater risk of liability deter industry from sponsoring neonatal trials [8].

## Regulatory framework for drug trials in India: a roller-coaster ride

Apart from the specific challenges in conducting drug trials in neonates, there are generic challenges that affect trials in any age group in India such as the regulatory framework that has witnessed considerable change (Supplementary information). The regulatory framework in India has been sort of on a roller-coaster ride swinging to different extremes and perhaps now in process of settling at a modest level.

Ethical conduct of a clinical trial involves a fine balance between protecting the interests of trial participants and enabling clinical research that addresses the health needs of the local population. From the late 1990s, India became a favorite research destination for global pharmaceutical companies due to the availability of a drug naive and diverse population with many disease conditions, skilled English-speaking researchers, quality research output at a lower cost, easy recruitment of participants, and timely completion of trials [9]. The country’s rather lax drug regulatory system also provided a favorable environment by facilitating faster trial approvals and poor oversight. This resulted in a large number of clinical trials that focused on testing drugs developed in Western countries or addressed health issues that were not needs-driven [10].

In 2012, women’s health activists filed a petition in the Supreme Court of India alleging ethical misconduct by sponsors and investigators in a human papilloma virus vaccine trial among Indian girls [11]. In 2013, the Central Drugs Standard Control Organization (CDSCO), directed by the Supreme Court of India, framed new regulations to protect the rights, safety, and well-being of clinical trial participants [12]. These included the accreditation of ethics committees, guidelines for the reporting of serious adverse events (SAEs) during clinical trials, and compensation for participants in cases of trial-related injury or death. After these regulations came into effect, approvals for new clinical trials decreased significantly and many foreign-funded trials moved out of India (Supplementary information).

The stiff regulatory requirements and lack of clarity on several aspects of the regulation made conducting regulatory trials a herculean task. The subsequent expression of great concern by the academic community and the pharmaceutical industry led to rationalization of the regulatory framework that has been gradually restoring a conducive environment for undertaking clinical trials [13–15].

We recently participated in a multi-centric regulatory “goat lung surfactant extract (GLSE)” trial [16]. The challenges faced by an investigator in conducting a regulatory clinical trial in a developing country are poorly described in the literature. In this article, we aim to highlight the difficulties we encountered in approval, implementation, and conduct of the GLSE trial, and put forward suggestions for streamlining these processes.

## The GLSE trial: challenges faced by the investigators

The GLSE was a multi-centric, double-blind RCT, designed and initiated by academic investigators without any conflict of interest and funded by Wellcome, UK. The Clinical Development Services Agency (CDSA), an initiative of the Department of Biotechnology (DBT), Ministry of Science and Technology, India provided the services of a contract research organization (CRO). This trial compared the safety and efficacy of a new surfactant (GLSE) with beractant (Survanta, AbbVie, USA) in preterm neonates with respiratory distress syndrome. The study results are being published in this supplement. (Datasets are available upon request from the corresponding author.) Here, we discuss the major issues and challenges faced by us during the conduct of this regulatory trial.

## Clinical trial approval and delays

All clinical trials investigating a new drug or new chemical entity in India require approval from the CDSCO, the Indian counterpart to regulatory authorities like the United States Food and Drug Administration (US FDA) and the European Medicines Agency (EMA). The CDSCO is headed by the Drugs Controller General of India (DCGI), who is also responsible for inspection of drug manufacturing facilities, trial sites, and sponsors.

The GLSE came under the category of an investigational new drug (IND) developed from a new source and not used in the country earlier. Since GLSE was developed in India, pre-clinical data had to be submitted to CDSCO and clinical trials of all phases (1–3) had to be carried out in India for regulatory approval. Since surfactants are administered into the trachea in intubated infants, conducting a phase-1 trial of GLSE among healthy volunteers was not feasible. Hence, the trial was designed as a combined phase-2 and phase-3 study in preterm neonates with RDS. The trial sponsor (All India Institute of Medical Sciences [AIIMS], New Delhi) submitted the trial application to the CDSCO and to the Institute Ethics Committees (IECs) of various participating sites. Table 1 lists the regulatory requirements for conducting a clinical trial in India.

**Table 1:** Requirements to conduct a regulatory clinical trial in India

1. Permission from Drug Controller General (India) The guidelines for the submission of application and conduct of regulatory trial in India are provided by the Drugs and Cosmetics Act of 1940 and its rules 1945, 122A, 122B, and 122D and the appendices of Schedule Y
2. Institute Ethics Committee (IEC) approval from all the participating sites
3. Health Ministry Screening Committee (HMSC) approval if funding is from outside India. This is a high-level committee housed at the Indian Council of Medical Research (ICMR), consisting of representatives from various health and administrative sectors and chaired by the Secretary, Department of Health Research, India. The committee meets once every 3 months to review trial applications and requires both IEC and CDSCO approval for processing
4. Foreign Contribution Regulation Act (FCRA) in case of foreign funding
5. Trial registration in the national registry (CTRI)

**Fig. 1:**
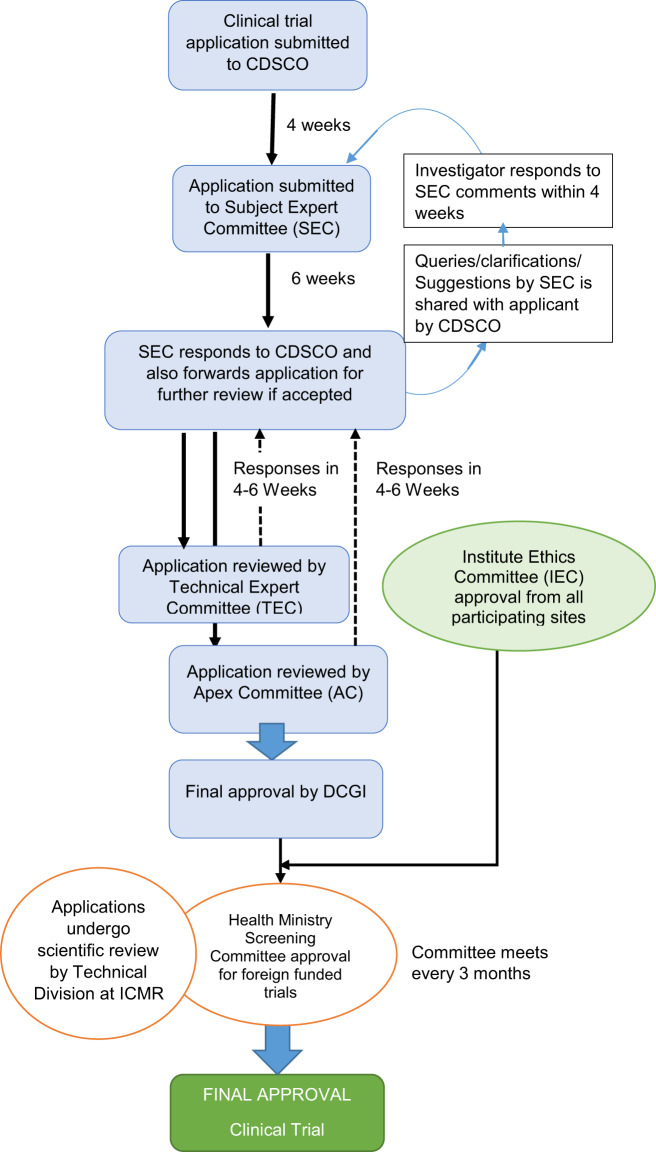
Process of regulatory approval for clinical trials in India

From 2013, the CDSCO follows a three-tier process to review trial applications: a first review by the Drug Advisory Committee consisting of subject experts followed by a second review by the Technical Committee, and finally by an Apex Committee (Fig. 1) [17]. The GLSE study being funded by a foreign agency, also required clearance from Health Ministry Screening Committee (HMSC) [18]. We submitted clinical trial application in November, 2013 and received final approval from all authorities for trial implementation in May 2016 (30 months). We discuss the factors that contributed to the delay in the approval of GLSE trial.

### Delay in ethical clearance

The IECs in a developing country like India face several challenges, including lack of experts with formal training in bioethics, non-scientific and legal experts who have limited experience with regulatory trials, infrequent meetings, and lack of clearly defined roles and responsibilities of its members [19]. The multi-center nature of the study, required that the trial be approved by each of the study centers’ Institute Ethics Committee (IEC), and this took time. There were differences between as well as disagreement within IECs in terms of trial-related risks to neonatal participants, the content and process of informed consent, etc. Lack of neonatal or pediatric subject experts in the IECs as well as members’ limited experience with regulatory trials also contributed to the delay.

### Health Ministry’s Screening Committee (HMSC) clearance- steps in series

Foreign-funded trials to be conducted in India require HMSC approval. However, applications are accepted only after approval from all IECs, as well as from CDSCO. It has its own expert review process and meetings happen once in 3 months (Supplementary information). Serial processes, after IEC and CDSCO clearance (rather than parallel review by HMSC) also contributed to delay in trial implementation.

## Informed consent and the issue of audio-visual (AV) recording

In 2013, the CDSCO mandated that investigators perform A-V recording of the informed consent process in addition to written consent in all clinical trials so as to ensure that the process of informed consent is not undermined in anyway. This was modified in 2015 to make A-V consent mandatory only in trials involving vulnerable populations (at risk of undermining the process of informed consent as in destitutes, military recruits, or medical/nursing students among many others) and when new chemical entities are studied [20].

Neonates and children are classified as vulnerable population who possess none or limited capacity for understanding information or making an informed decision [21]. Investigators need to obtain written informed consent from parents or legally authorized representative (LAR) without coercion or undue influence. The infant’s parents or their LAR indisputably act in the best interest of their ward and they cannot be considered vulnerable. The process of A-V recording of consent in addition to written informed consent is time consuming and requires specific infrastructure [22]. Thus, this requirement of mandatory A-V consent may deter investigators from doing trials involving neonates. Secondly, neonates in an intensive care unit are critically ill and parents are often approached at a stressful time with complex information about the study. In an emergency situation such as surfactant administration, AV- recording is practically impossible and hence we requested IECs and regulatory authorities to waive off A-V recording. As the situations where A-V recording can be waived off have not been clearly specified, we had hard time obtaining the waiver. One IEC refused to offer this waiver and this precluded the center’s participation in the trial.

## Safety reporting and causality assessment

**Fig. 2:**
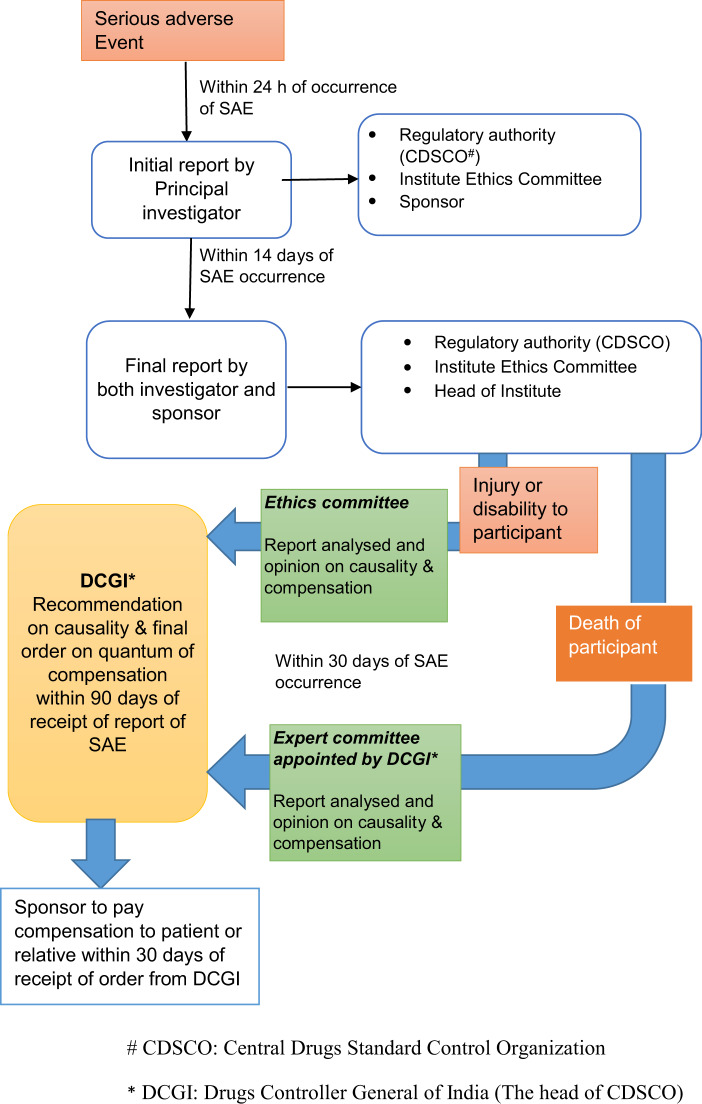
Process of reporting serious adverse events (SAE) during a regulatory clinical trial in India. CDSCO: Central Drugs Standard Control Organization, DCGI: Drug Controller General of India

Investigators are required to report all SAEs within 24 h of their occurrence to the sponsor, the regulatory authority (CDSCO) and to the IEC. This is followed by a detailed follow-up report on SAE independently by the investigator and the sponsor to the head of the Institution (of the trial site), CDSCO and to the IEC within 14 days of known occurrence of SAE (Fig. 2). IECs analyze all SAEs and forward its report along with opinion on financial compensation, if any, to be paid by the sponsor to the DCGI within 30 days of occurrence of SAE. All SAEs are reviewed by CDSCO and the final adjudication is communicated to the investigators. When the SAE is death or permanent disability, the CDSCO constitutes an independent expert committee to arrive at the cause of death and to recommend the quantum of compensation within 30 days of receiving the report from expert committee in accordance with the formula specified in the Seventh Schedule [23, 24].

Certain points regarding SAE reporting and compensation require discussion.

### Reporting of SAEs

An adverse event (AE) is any untoward sign, symptom, diagnosis, or abnormal laboratory parameter that occurs in a participant enrolled in a trial. An adverse event that leads to death, permanent disability, or hospitalization of trial participant is labeled as a SAE. Timely and transparent reporting of all SAEs is essential to ensure safety monitoring of new interventions. Critically ill preterm neonates are at high risk of morbidity (like nosocomial sepsis, apnea, anemia, necrotizing enterocolitis, bronchopulmonary dysplasia) or have abnormal laboratory reports that fall under the purview of SAE. We reported 246 SAEs in 98 participants in GLSE trial. Thus, it is challenging for a clinician to identify, interpret, label, and report a substantial number of SAEs. For example, an adverse event is upgraded as SAE if it results in prolongation of hospitalization. However, defining prolonged hospitalization is difficult as the duration of hospital stay in a neonate is variable depending on gestational age and other co-morbid factors.

### Causality assessment of SAEs

There is lack of general agreement on the process of causality assessment of SAE. The World Health Organization Collaborating Center for International Drug Monitoring-the Uppsala Monitoring Center (WHO-UMC) [25] and the Naranjo probability scale [26] are the two most common tools used in adults but these are not appropriate for use in neonatal population or in hospitalized subjects. Also, there are no clear recommendations for investigators whether, individual SAEs that occur together or as a chain of events secondary to one underlying disease process need to be reported and analyzed for causality as one SAE or individually. For example, let us consider a hypothetical example of a neonatal participant enrolled in a trial. This neonate develops apnea at 16 h of life due to sepsis, sclerema at 36 h, shock at 44 h, and pulmonary hemorrhage at 52 h of age. The blood culture is reported as positive at 72 h of age and the infant dies at 90 h of age. Several SAEs (apnea, sclerema, shock, pulmonary hemorrhage, culture-positive sepsis) and death are observed in this case—all due to a single disease pathology, namely sepsis.

For the GLSE trial, we prepared a guidance document approved by all site investigators to streamline and maintain uniformity of reporting SAEs (Supplementary information). We described possible AEs for the study and categorized them into severity grades (mild, moderate, severe, life threatening, or resulting in death). Investigators were to report only severe and life-threatening AEs as SAEs.

### Free medical care for trial-related injuries

The sponsor is required to provide free medical care for all trial-related injuries in the participant as long as required or till such time it is established that the injury is not related to the clinical trial, whichever is earlier. Adjudication and establishing causality of an SAE takes time. As most sick neonates experience one or more SAEs, this translates into providing free care to practically all enrolled in a trial. While this is possible at public hospitals (as the treatment is anyway free for all patients), it is quite challenging at private hospitals.

Trial sponsors are required to submit financial plans including insurance for providing free medical care for SAEs and monetary compensation where required. This financial burden is huge in trials involving sick and preterm neonates and may not be borne by a single sponsor alone (especially in academic trials). These issues precluded many private hospitals from participating in the GLSE trial.

### Quantum of compensation for trial-related injury or death

In addition to free medical treatment, the sponsor is required to pay financial compensation for permanent injuries or death causally related to the trial. In 2013, drugs and cosmetics rules were amended (known as Rule 122 DAB) to address the quantum of financial compensation in clinical trials (Table 2) [23, 24]. As per the new regulation, the compensation for trial-related death is calculated using the formula:

$${\mathrm{Compensation}} = \left( {B \ast F \ast R} \right)/99.37$$

Where, *B* = base amount, i.e., 800,000 INR, *F* is factor depending on the age of the subject, and *R* is the risk factor depending on the seriousness and severity of the disease, presence of co-morbidity, and duration of the disease of the subject at the time of enrollment in the clinical trial. The base amount is calculated such that the nominee of the participant gets an amount equivalent to the minimum wages of the unskilled worker in New Delhi as interest, if the amount of compensation is kept as fixed deposit in a bank. The factor (*F*) varies from 228.54 (<16 years) to 99.37 (>65 years) and the risk factor varies from 0.5 to 4.0 on the basis of the participants’ clinical condition. For instance, a risk factor of 0.5 is chosen for a terminally ill patient who is not expected to survive >6 months, 4 for healthy volunteers or participants without risk and 1, 2, or 3 for those with high, moderate, or mild risk of mortality. Even in cases where expected mortality is 90% or more within 30 days, a fixed amount of INR 200,000 should be given [23].

**Table 2:** Compensation for clinical trial-related injury and death to participants

Serious adverse event	Formula for deriving the compensation amount	Parameters
Death	(*B* × *F* × *R*)/99.37	*B* = Base amount, i.e., 800,000 INR *F* = Factor depending on the age of the subject *R* = Risk factor depending on the seriousness and severity of the disease, presence of co-morbidity and duration of the disease of the subject at the time of enrollment in the clinical trial between a scale of 0.5 to 4
Permanent disability to subject	(*D* × 90 × *C*) /(100 × 100)	*D* = Disability percentage. *C* = Compensation amount for payment to the participant’s nominee(s) in case of death of the participant
Congenital anomaly/ birth defect	*B*/2	*B* = Base amount, i.e., 800,000 INR
Life-threatening disease	2 x *N* × *W*	*N* = Number of days for life-threatening situation requiring medical care, irrespective of days of hospitalization *W* = Minimum wage per day of the unskilled worker (in Delhi)

Thus, it is clear that both *F* and *R* values cannot be translated to the neonatal population as the criteria provided are not suited in context to the neonates. There are no guidelines for assigning the value for risk factor or age factor for neonates. The variable course of neonatal illness and future neurodevelopmental outcomes make prediction of permanent disability difficult.

Using the formula recommended by CDSCO, the minimum and maximum financial compensation in the event of trial-related death of a neonate works out to be 9.2- and 73.6-hundred thousand INR, respectively. Even the minimum amount is huge and parents from financially disadvantaged backgrounds may be unduly influenced and volunteer to participate in a study for sheer financial gains (inducement: potentially compromising the spirit of free and voluntary participation). On the other hand, this may deter investigators and sponsors from conducting regulatory trials because of the substantial cost and difficulties in arranging funds [27].

For the GLSE trial, we prepared a guidance document for deciding compensation for trial-related injury (Supplementary information). After detailed discussion and considering high mortality in the subjects without intervention, the risk factor was decided as 0.5 to be used to calculate compensation in the study.

### Clinical trial insurance

All medical care and compensation for trial-related injury are borne by the sponsor. Obtaining health insurance coverage for clinical trials is a great challenge especially if the trial sample size is large, involves a high-risk population, for specific drug types or interventions or when adverse events are anticipated. In such trials it is difficult to calculate insurance amount, premium, and actuaries. In GLSE trial, insurance companies were reluctant and it required a great deal of persuasion to obtain insurance cover.

## Operational challenges

We describe specific operational challenges of implementing the GLSE trial and how we successfully navigated these in a resource limited setting.

### Site selection and engagement

The study sites varied in investigator experience, ability to recruit adequate number of participants within the desired time frame, infrastructure, and manpower. Dedicated research staff (one medical doctor and four nurses) were recruited at all participating sites for round-the-clock enrollment of participants. The staff were trained by the coordinating center (AIIMS) and CDSA on the study protocol, standard operating procedures (SOP), and Good Clinical Practices (GCP). While availability of nurses for research jobs is plentiful but that of physicians is pretty limited. The lack of requisite expertise among research staff pose serious challenge requiring a lot of training and supervision.

### Quality management

To ensure compliance with GCP standards, quality control and quality assurance measures were put in place. The CDSA provided support services including on-site trial monitoring, regular remote monitoring, and capacity building. The monitors were trained on clinical aspects of the trial and they verified the handling and storage of investigational product, consent process, patient allocation, data capture, and compliance with the trial protocol.

### Trial data management

Data were captured first in paper case record form (CRF) and then transcribed onto the electronic CRFs (eCRFs) at sites. The option of an exclusive real-time electronic data capture was not feasible due to inconsistent internet connectivity at a few sites and lack of option of offline data entry in the database platform used in the trial. The commercial international electronic database platforms (~250-thousand dollars) hugely exceeded our trial budget [28]. We opted for an Indian database platform, which had reasonable capabilities but certain limitations requiring considerable troubleshooting during the trial. The CDSA staff assisted the research team in periodic troubleshooting, data entry, query generation, and query resolution. The high standard of data quality of the GLSE trial received appreciation from the data safety monitoring board (DSMB) and Research Steering Group.

### Enormous paperwork

Obtaining written informed parental consent, reporting, and follow-up of SAEs required documentation in the form of narratives. It was challenging to ensure high quality and timely submission of these voluminous narrative reports to the regulatory authorities. The narratives added to the burden of documentation at the sites, but since it was a regulatory trial, redundancy of documentation was preferred over deficient documentation.

## New regulatory changes

In March 2019, the CDSCO notified the new Drugs and Clinical Trial Rules, 2019  [23], in the official Indian gazette. These rules pertain to all new drugs, investigational new drugs for human use, clinical trial, bioequivalence study,bioavailability study and Ethics Committees. Few important changes are discussed below. Academic clinical trials require only IEC approval and no longer need DCGI permission. In an academic clinical trial, an investigator studies a drug already approved by the DCGI (for a specific indication), for a new indication, new route of administration or new dose or dosage form. However, the results of academic trial should not be used for marketing the drug for new indication. The CDSCO has also introduced fast tracking of review and disposal of clinical trial applications (CTA) of a new drug. If the trial drug is discovered, researched, and developed in India with a proposal to manufacture and market in India, the time period of process of application has been shortened to 30 days. In case of the trial drug is already approved and marketed in countries specified by CDSCO, then the maximum time limit for CTA is 90 days. All clinical trials need mandatory prospective registration with the clinical trial registry of India (CTRI) before enrollment of first patient. The trialists are also required to submit six monthly status report of each regulatory clinical trial, to the CDSCO electronically via the on-line SUGAM portal. The new rules also waive off the need to conduct a phase III clinical trial of a new drug in the Indian population, if the drug has been already been approved and marketed in selected developed countries. However, they still need to conduct a phase IV trial after the drug has been marketed in India to evaluate the long-term effects. The DCGI can also exempt drugs used for diseases that have unmet need or have special relevance to Indian health scenario and orphan drugs (which treat conditions affecting less than 500,000 Indians), from both phase III and IV clinical trials.

## The way forward

We have summarized the many logistical and operational challenges faced while implementing a regulatory trial of a new drug among neonates in a low-resource setting. We now, also put forth a set of recommendations, which we believe would promote a congenial environment for smooth conduct of clinical trials in India. These recommendations call for multi-level interaction and involvement of all stakeholders (the investigators, sponsors, regulators, health care policy makers, administrators, and patients) to contribute their knowledge, capacities, and structured feedback for fair conduct of clinical trials.

### Development of national clinical trial infrastructure

Researchers in a low- middle- income (LMIC) set-up need support and training to conduct clinical trials on their own or collaborate with other researchers. Creation of national level clinical trial infrastructure that provides investigator training, experienced clinical trial personnel, offers protocol development support and assistance with regulatory submission, quality control system, data-management support, SAE reporting, etc. would be important. The establishment of clinical trial networks or consortia funded by government exist in many countries [29] and have led to investigator training in clinical trials and good clinical practices, higher participant recruitment, and even attract funding from sponsor agencies. A web repository of clinical trial resources would assist the trialists a great deal.

### Regulatory approval

The clear and unambiguous stipulation of regulatory provisions that applies to diverse situations would go a long way in efficient approval of the trials. The importance of educating different stakeholders, including CDSCO functionaries, IEC members, academicians, CROs, industry people cannot be overemphasized. The conditions when the A-V recording of consent can be waived off needs to be specified. Development of a web-based portal for clinical trial application with a common platform where regulators, sponsors, investigators, and IECs can access the status of clinical trials using unique trial IDs can increase transparency, efficiency in trial approvals and encourage various authorities to work in parallel. Avoidance of redundancy in review process (relevant experts in one committee rather having multiple rounds of reviews; pharmacology experts can be opted in CDSCO committees rather than having a separate ICMR review for new drugs) would be greatly helpful. Some efforts by CDSCO in this regard include the on-line SUGAM portal for trial registration and provision of a pre-submission and post submission meetings with investigators/sponsors who seek clarification in person in respect of new trial application or queries related to pending application [30].

### HMSC approval

Foreign-funded trials, seeking HMSC approval needs faster disposal. The review process can work in parallel to other approval processes. There should be situations when the approvals can happen at a lower level rather than all applications being considered by full board only.

### Functioning and training of ethics committees

For multi-center trials, approval by a centralized ethics committee or IEC attached to nodal center can result in faster approvals. The serial and independent review of a single protocol at multiple IECs, leading to different queries results in significant loss of time and resources. India can take the example of Single Institutional Review Board (IRB) policy recently followed by National Institute of Health in United States [31]. According to this policy, one participating site serves as the single IRB and provides ethical review for the common research protocol for all domestic sites participating in a multi-site study. The IEC members require both initial and continuing training on research ethics, methodology, and regulatory guidelines [32]. They also require training in SAE analysis, causality assessment and monitoring of trial implementation.

Training and capacity building of IECs has been shown to positively impact the knowledge of both the trained and untrained IEC members and enhance the functioning of IECs [33]. In this regard, the Forum for Ethics Review Committees in India (FERCI) has established a network of ECs and has contributed to capacity building of its members through various initiatives. One such initiative is the creation of software tools to facilitate the functions of IEC. It has also trained over 30 IECs, which are now called “Smart IECs” [34]. While, reviewing a trial involving neonatal or pediatric population, IECs should mandate the participation of a pediatrician or neonatologist as member or as a special invitee. This would help the IECs to understand the trial and its implications better.

### Informed consent

Audio-visual recording of consent process might prove difficult in emergency situations and regulatory authorities should consider waiving A-V recording in such legitimate situations. The current regulatory framework does not address consent in emergency situations and offers no guidance on alternatives like prospective consent or consent waiver. It might be appropriate for IECs and regulators to consider trials involving such scenarios on a case by case basis and evaluate the feasibility of alternatives to allow timely completion of studies. Investigators need to consider infrastructure and additional funding for A-V consent recording when planning trials.

### SAE reporting

Reporting of SAEs can be streamlined by using an electronic or web-based platform for notifying regulators, IECs, and Sponsors. Follow-up detailed reports could similarly be updated in the same platform using a uniform template. All IECs should have sub-committee for SAE review and causality assessment. To standardize causality assessment of SAEs and reduce subjective variation among investigators, standardization of SAE classification for neonatal clinical trial would be of help.

### Compensation for trial-related death or injury

The quantum of compensation for trial-related injury/death among neonatal participants need to be adapted for neonatal context. The value of “*R*” that is factored in the calculation of compensation should be adapted for severity of neonatal diseases, presence of risk factors, including prematurity and co-morbid conditions like bronchopulmonary dysplasia, asphyxia, etc. at the time of enrollment in the clinical trial. Also, authorities and investigators need to agree on the length of follow-up of neonates to determine the degree of disability or neurodevelopmental impairment.

### Clinical trial insurance cover

There is an urgent need for guidelines that dictate the principles and procedures for insurance coverage, which the sponsor of a clinical trial must provide to protect trial participants. This includes whether the insurance coverage would include all the costs associated with investigations and treatments that arise from participation in the trial, the limit and period of insurance coverage, liability per claim and the aggregate liability, etc. The IECs and other stakeholders must be educated to critically examine insurance policies submitted for approval.

### Funding

Funding for clinical trials is difficult in LMIC set-up and generally comes from developed countries or pharmaceutical companies. Governmental agencies, academic groups, voluntary health organizations as well as industry should come forward to support need-based research that address health problems of the country. Another solution to reduce costs and achieve faster trial completion is to incorporate multiple trial designs (either sequentially or in parallel) on a single large platform and opt for multi-sponsor financial support [35]. Initiatives that can foster research culture in the country include providing grants and fellowship schemes, upgrading academic infrastructure, promoting research networks. and facilitating funding mechanisms. Availability of free and open-source data-management system for clinical trials that adheres to evolving standards such as those set by the Clinical Data Interchange Standards Consortium (CDISC) would significantly decrease the costs associated with electronic data capture in LMIC settings.

## Conclusion

Researchers in academic institutions engaging in investigator-initiated clinical trials face several challenges in obtaining regulatory approval and in conducting the trial with limited resources. For trials involving neonates, the requirement for audio-visual recording of consent process, financial planning for free medical care and monetary compensation for treatment trial injury/death are particularly challenging in India. The creation of national clinical trial infrastructure and research networks could provide much needed support to investigators. The Indian scenario is witnessing a paradigm change with new regulations and capacity-strengthening initiatives of research ethics committees. These initiatives can foster more need-based research by providing a congenial research environment while at the same time protecting the rights and interests of study participants.

## Disclaimer

The views expressed in the article belong to the authors in their individual capacity and do not reflect in anyway the opinions and policies of the institutions they belong to.

## Supplementary information

Supplementary Information
