# Supplementary material for: Issues, challenges, and the way forward in conducting clinical trials among neonates: investigators’ perspective
Source: J Perinatol. 2019 Sep 4;39(Suppl 1):20–30. doi: 10.1038/s41372-019-0469-8 (PMC8075906; doi:10.1038/s41372-019-0469-8)
Supplement: Supplementary file 1 — Supplementary Information [file 41372_2019_469_MOESM1_ESM.docx]

**Supplementary Information for “Issues, challenges and the way forward in conducting clinical trials among neonates -Investigators’ Perspective”**

**Index**

Figure S1: Timeline of changes in regulatory scenario in India from 1940 till 2018. In September 2013, the Central Drugs Standard Control Organization (CDSCO) introduced several amendments in the Schedule Y of drugs and cosmetic rules

Figure S2: Timeline of regulatory changes pertaining to clinical trials in India respect to international scenario

Figure S3: The graph shows the number of clinical trials approved by Central Drugs Standard Control Organization (CDSCO) before and after 2013 in India.

Figure S4: Health Ministry Screening Committee (HMSC) approval process in India

S1: Adverse Events (AE) with Severity Grades definitions used in GLSE trial

S2: Guidance document for deciding compensation for trial-related injury in GLSE trial

**Figure S1:** Timeline of changes in regulatory scenario in India from 1940 till 2018. In September 2013, the Central Drugs Standard Control Organization (CDSCO) introduced several amendments in the Schedule Y of drugs and cosmetic rules.

Abbreviations: A-V: Audio-visual recording, CDSCO: Central Drugs Standard Control Organization, CTRI: Clinical Trial Registry of India, DCGI: Drug Controller General of India, ICMR: Indian Council of Medical Research, IEC: Institute Ethics Committee.

**Figure S2: Timeline of regulatory changes pertaining to clinical trials in India respect to international scenario**

*Indian scenario:* In 1940, the Drugs and Cosmetic Act was formulated to regulate the import, manufacture and distribution of drugs in India. There were no guiding principles for ethical conduct of research in human subjects until the Indian Council of Medical Research (ICMR) published a policy statement on ‘ethical considerations involved in research on human subjects’ in 1980. In 2005, Drugs and Cosmetic Act was modified to include guidelines for drug trials. Registration with clinical trials registry of India (CTRI) became mandatory in 2009. Stricter guidelines for clinical trials were formulated only in 2013 instigated by allegations on the conduct of human papilloma virus vaccine trial on adolescent girls in India. In 2013, DCGI made three-tier system of screening clinical trials, rules for reporting serious adverse events and compensation.

*International scenario:* Research ethics started after World War II in 1946 condemning the medical experiments conducted on prisoners without their consent. As a result, Nuremberg Code was established in 1948, which mandated voluntary participation and informed consent for any clinical trial. In 1964, World Medical Association developed Declaration of Helsinki as ethical principles for medical research involving human subjects. When the Tuskegee Syphilis Study (1932-1972) was made public, it led to nationwide protests terminating the study. Following this, National Commission drafted the Belmont Report that summarized the basic ethical principles and guidelines for the conduct of research in human subjects. In 1990, International Council for Harmonization brought regulatory authorities from US, Europe and Japan together. Common rule is a set of regulations for research involving human subjects that have been adopted by many countries.

Code of Federal regulations (CFR) issued by Federal Drug Administration (1981)

Declaration of Helsinki (1964)

The international council for Harmonisation of technical regulations for Pharmaceuticals for human use (ICH)

Belmont report by National Commission (1979)

Nuremberg code (1948)

Drugs and Cosmetics Act (1940)

ICMR publishes ethical consideration for research involving human subjects

Revision of ethical guidelines by ICMR

Schedule Y of Drugs and Cosmetics Act amended to include guidelines for drug trials

Trial registration with CTRI made mandatory

Three new rules introduced in schedule Y; 122 DAB, 122DAC and 122DD

New clinical trials rule, 2018

Drugs and Cosmetics Act amended

Audio-Visual consent only for vulnerable participants, 3 trials per investigator cap revoked, “SUGAM” portal for online trial application

Registration of IECs with DCGI made mandatory

Abbreviations: CTRI: Clinical Trial Registry of India, ICMR: Indian Council of Medical Research, IEC: Institute Ethics Committee, DCGI: Drug Controller General of India

**Figure S3:** The graph shows the number of clinical trials approved by Central Drugs Standard Control Organization (CDSCO) before and after 2013 in India.

**Figure S4:** Health Ministry Screening Committee (HMSC) approval process in India

All research proposals involving foreign funding or international collaboration require approval from Government of India’s (GoI), Health Ministry’s Screening Committee (HMSC). The ICMR is the secretariat of Health Ministry’s Screening Committee (HMSC).

**Composition:** HMSC is chaired by Health Secretary with alternate chairman Director General-ICMR and other representative members from Ministry of Science and Technology, Department of Biotechnology, Ministry of External Affairs, Armed Forces Medical Services and Ministry of Finance. The ICMR reviews the scientific and technical content of the project and forwards it to HMSC. The HMSC meets after 3-4 months on an average. The HMSC may approve or reject the project or ask for additional clarifications. It can take a minimum time of 6-8 months from the time of project submission to final approval. The HMSC requires Institutional Ethics Committee (IEC) clearance of all the participating centres sites at the time of submission of the proposal to ICMR. For clinical drug trials, clearance from office of Drugs Controller General of India (DCGI) is also required and the trial needs to be registered with the Clinical Trial Registry in ICMR (CTRI). The project is likely to be deferred by HMSC in the absence of DCGI and or IEC clearance certificate.

# **S1: Adverse Events (AE) with Severity Grades definitions used in GLSE trial**

Adverse is defined as any unfavorable and unintended sign (including an abnormal laboratory finding, for example), subjective and objective symptom, or disease temporally associated with the use of a medicinal product, whether or not considered related to the medicinal product. This includes an exacerbation of pre-existing conditions or events, or drug interaction or significant worsening of the disease under investigation that is not recorded elsewhere in the CRF under specific efficacy assessments.

The Investigator needs to record AEs in AE Log provided in the CRF. In addition, they need to assess the intensity of adverse event and grade accordingly as described below:

1. **Mild:** asymptomatic or mild symptoms; clinical or diagnostic observations only; intervention not indicated.
2. **Moderate:** minimal, local or non-invasive intervention indicated
3. **Severe:** Severe or medically significant but not immediately life-threatening; hospitalization or prolongation of hospitalization indicated; disabling.
4. **Life Threatening:** Life-threatening consequences; urgent intervention indicated.
5. **Death**

The AEs that fulfils criteria for SAE must be reported to regulatory authorities as per the timeline and format specified. This document provides guidelines as to how to assign intensity level to a given AE. This document provides a broad guidelines and the investigators are advised to exercise their judgment for an individual case.

**Guidelines**

- SAE reporting should be done for the adverse events (table below) that fall into ‘severe’, life-threatening’ categories (categories 3 & 4) or death.
- SAE reporting should preferably involve reporting of a disease and not a sign or symptom. However, if the underlying disease is not obvious when a sign or symptom appeared, the later can reported. Once a disease is reported and the baby manifests another sign or symptom related to the reported disease, there is no need to report again that sign or symptom as SAE. However, the all the sign/symptom/lab abnormality/worsening of severity of disease have to be entered in AE log in CRF.
- When an SAE occurs, initial notification must go within 24 hours. The window of 24 hours provides opportunity for clinical work up of the patient to know the underlying disease. If the clinical team is able to ascertain with a reasonable confidence, they can the underlying disease and there is no need of reporting symptom. If the cause of sign/symptom is unknown, the same should be reported within 24 hours.

**Examples**

1. A 30-wk baby developed apnea at 16h of life due to sepsis. Later, the baby developed sclerema at 36h, shock at 44h and DIC and pulmonary hemorrhage at 52h of age. The blood culture was reported as positive at 72h of age. The baby died at 90h of age.

- Apnea due to sepsis is not an SAE (see table below), therefore do not notify it as SAE. However, enter apnea in AE log.
- At 36 hours, sepsis worsened and manifested as sclerema, which is a life threatening condition, and therefore sepsis now qualifies for being SAE. Notify it as an SAE (severe sepsis, manifested as sclerema). Subsequent evetns namely shock, DIC and pulmonary hemorrhage, despite being having severe AEs, are actually the manifestations of the same disease (severe sepsis) already notified and therefore would not require fresh notification. However, enter these events in the AE log and specify the severity as per the table.
- Death being an extreme event must be always be notified within 24 hours of its occurrence (even if it is due to a disease already notified).
- Detailed report must contain description of all the events including apnea, sclerema, shock, pulmonary haemorrhage and culture positivity with outcome being death.
  - The scanned copy needs to be shared with Medical Monitor (MM, CDSA) for checking completion and accuracy by the 7^th^ day.
  - After incorporating feedback of MM, the hard copy of the detailed report must be shared with AIIMS within 10 days of occurrence of SAE.

1. A 26 wk/ 780g baby born to a mother with chorioamnionitis developed shock at 5h of life. The baby is started with antibiotics and ionotropes but develops pulmonary hemorrhage by 12h dies at 15h of age.

- Since the time gap between events is <24h, notify only death with brief account of trail of events. Explanation of all the events can be provided in the detailed report.

1. The baby of 29 wk/860g was born to a mother with PIH. The baby received IP at 13h and a repeat dose at 25h of life. The baby developed clinically significant PDA on day-1 requiring pharmacological intervention. The baby developed shock on day-2 requiring inotropes and later right sided pneumothorax which worsened baby’s condition. The baby did not respond well to the treatment and died on day-4.

- Since PDA required medical management and not surgical intervention, it is not an SAE and need not be notified.
- Shock and pneumothorax qualify as SAEs and therefore require notification as two separate SAEs (both can be sent in a single email).
- Death must be notified within 24 hours (even if it is due to a disease already notified).
- As all SAEs occurred within a timeframe of 7 days, all of them can be clubbed in a single detailed report which needs to be shared by email with CDSA Medical Monitor (within 7 days) and hard copies with Sponsor and IEC (within 10 days).

1. A 27 wk baby was born to a mother with chorioamnionitis (high vaginal swab was positive for *Klebsiella pneumonia).* The baby developed RDS and received IP at 6h and a repeat dose at 13.5h life. The baby was diagnosed as sepsis based on maternal risk factors (before consent and enrollment) and was started on. The baby developed shock due to sepsis requiring inotropes on day-1 to day-3. On day-9, there was respiratory worsening requiring significant augmentation in ventilator support. The chest X- ray showed right sided infiltrates suggestive of ventilator associated pneumonia and the antibiotics were upgraded. The baby also developed anemia from day-2 to day-52 requiring three packed red blood cell transfusion. The baby developed PDA on day-9 requiring IV paracetamol.

- Sepsis occurred before enrollment and need not be notified. Also, it is not an SAE.
- Septic shock requiring vasopressor (even when sepsis started before enrollment) and anemia requiring transfusion are SAEs: requires notification as two separate SAEs (in a single email).
- Since these 2 SAEs occurred within a timeframe of 7 days, they can be clubbed in a single detailed report, which is shared by email with CDSA Medical Monitor (within 7 days) for her feedback and hard copies with Sponsor and IEC (within 10 days).
- Though anemia resolved on 52^nd^ day, there was no change in causality or worsening, there is no need for follow up report. However, check with IEC if they want any follow-up report on resolution of an SAE.
- PDA required only pharmacological intervention is not an SAE.
- Sepsis worsened day-9. As the detailed report is already sent, it requires a follow up report.

1. A mother delivered at 28 wk gestation. The baby had asphyxia and required positive pressure ventilation. The baby developed RDS and received IP by 2.5h of life. On day-1, the clinical team suspected sepsis. Sepsis screen was positive but blood culture was negative. The baby was treated with IV antibiotics and recovered. Ultrasound of brain on day-2 revealed right sided P/IVH grade II. There were no symptoms associated with it. There was no evidence of post-haemorrhagic ventricular dilatation in follow up scans.

- Two events - sepsis and P/IVH are not SAEs and need not be notified. However, enter them in AE log.

1. A baby of 29 wk gestation was born to a mother with prolonged duration of rupture of membrane (72h). Her high vaginal swab was positive for Pseudomonas about 15 days prior to delivery for which she was treated with antibiotics. The baby developed respiratory distress and received IP within 6 hours of birth. The baby developed recurrent apnea at 90 h and required nasal IPPV. The baby developed abdominal distension with bilious aspirates suggestive of sepsis on day-4. The blood culture grew methicillin resistant *Staphylococcus aureus* (MRSA). The baby was treated with antibiotics. In view of thrombocytopenia and worsening level of sickness, antibiotics were further augmented. The baby was given packed red blood cell transfusion. The baby developed massive pulmonary hemorrahage and shock on the 5th day of life requiring ionotropes, morphine, and platelets and fresh frozen plasma. The baby died on day 6 of life.

- Apnea can be entered in AE log.
- Sepsis needs to be notified within 24 hours of blood culture coming positive.
- As shock, thrombocytopenia and pulmonary hemorrhage are the manifestations of sepsis and thus need not be notified within 24 hours but need to be described in the detailed report. They can be entered in AE log.
- Death being an extreme event must be notified within 24 hours of its occurrence (even if it is due to a disease already notified).
- Since the SAE’s occurred within a timeframe of 7 day, all of them can be clubbed in a single detailed report, which needs to be shared by email with CDSA Medical Monitor for her feedback (7 days) and to the Sponsor and IEC (10 days).

1. A 26 week baby enrolled and received IP within 2 h. the baby responded to it and was extubated and he remained on CPAP. However, the baby required mechanical ventilation for recurrent apnea on day-7. Baby was suspected to have sepsis-pneumonia and treated with antibiotics. Later, the baby also developed recurrent episodes of seizures resulting in hemodynamic instability requiring multiple vasopressors and hydrocortisone. There was no meningitis, IVH, hypocalcemia or electrolyte abnormality. The baby also had abdominal distension and bilious aspirates. Surgical consult did not suggest any surgical pathology. The cultures were sterile. The baby was treated with antibiotics for a total of 21 days. The baby finally recovered and extubated. The clinical picture of this baby was suggestive of late onset sepsis. However, the cause of seizures could not be ascertained and was possibly due to combination of factors like hypoxia and acidosis triggered by multiple episodes of apnea and shock. The baby also developed anemia requiring multiple transfusions from day-10 to day-20 of life.

- Recurrent apnea (due to sepsis) needs to be notified within 24 hours though, the apnea was due to culture negative sepsis but it required mechanical ventilation and therefore qualifies as SAE.
- As the cause for seizures was not obvious but it fulfills SAE criteria therefore requires notification. Shock happened due to multiple episodes of seizures and therefore need not be notified as separate SAE (despite this fulfilling criteria for SAE).
- Anemia requiring PRBC transfusion needs to be notified within 24 hour.
- Detailed report must go to Medical Monitor by day-7 of the initial SAE (recurrent apnea) and Sponsor and IEC by day 10. The SAEs that could not be included in first detailed report need to be included in 2^nd^ deatiled report.

1. A baby of 32-wk gestation was born to a mother with beta thalassemia and severe. The baby developed respiratory distress and received IP at 1h and repeat dose at 13. The baby developed polycythemia (haematocrit 70%) on day 1 of life and required partial exchange transfusion. The baby developed clinical sepsis on 4^th^ day of life. The sepsis screen was positive, CSF was normal and culture was sterile (late onset culture negative sepsis). The baby was treated with antibiotics for 8 days. The baby also developed hyperbilirubinemia requiring phototherapy on day-5.

- All the events (polycythemia, culture negative sepsis, hyperbilirubinemia) do not satisfy the SAE criteria and thus need not be notified initially within 24 hou

| **S. N** | **Adverse Event** | **Grades** | | | | |
| --- | --- | --- | --- | --- | --- | --- |
|  |  | **1 (Mild)** | **2 (Moderate)** | **3 (Severe)** | **4 (Life-threatening)** | **5 (Death)** |
|  |  |  |  | **(To be reported as SAE)** | | |
|  | Apnea |  | - Apnoea needing methyl xanthine therapy or non-invasive respiratory support | - Need of invasive respiratory support |  | - Death |
|  | Definition: A disorder characterized by cessation of breathing for at least 20 seconds or accompanied by bradycardia | | | | | |
|  | Air leaks including pulmonary interstitial emphysema(PIE) |  | - Minimal free air in the pleural cavity NOT requiring increase in respiratory support, OR - PIE | - Free air in the pleural cavity requiring increase in respiratory support or drainage or need of high frequency ventilation | - Tension pneumothorax, OR - Bilateral pneumothorax resulting in circulatory compromise | - Death |
|  | Definition: A disorder characterized by presence of free air in the pleural cavity | | | | | |
|  | AKI/ARF | - | - Serum creatinine increase of >=0.3 mg/dL within 48 h OR - 1.5-1.9 times the lowest previous value within 7 days OR - Urine output (ml/kg/h): <0.5 for 12 h | - Serum creatinine: 2-2.9 times the lowest previous value OR - Urine output (ml/kg/h): <0.5 for >=12 h | - Serum creatinine: >=3 times the lowest previous value OR - >=2.5 absolute value - Urine output (ml/kg/h): <0.3 for >=24 h OR anuria for >=12 h - OR need of dialysis | - Death |
|  | Definition: A disorder characterized by - Sudden impairment in kidney function that results in the inability to maintain adequate fluid, electrolyte, and waste product homeostasis | | | | | |
|  | Arrhythmias | - Incidental detection, transient and NOT causing cardiac shock and NOT needing any therapy | - Asymptomatic but need of medical therapy | - Symptomatic causing cardiac failure and need of medical therapy | - Symptomatic needing cardio version or surgical therapy | - Death |
|  | Definition: A disorder characterized by abnormal heart rhythm diagnosed by ECG | | | | | |
|  | Anemia | - Asymptomatic anemia NOT needing blood transfusion |  | - Anemia needing blood or exchange transfusion |  |  |
|  | Anemia: A disorder characterized by decreased hemoglobin in blood | | | | | |
|  | Aspiration syndromes |  | - New infiltrated or shadows in chest X-ray along with need of oxygen therapy by hood or nasal cannula | - New infiltrates or shadows in chest x-ray requiring invasive or non-invasive ventilation or increased ventilation requirements | - New infiltrates or shadows in chest x-ray along with increased oxygen or ventilation requirements resulting in need of high frequency ventilation due to hypoxia or hypercarbia | - Death |
|  | Definition: A disorder characterized by aspiration of milk, blood, vomitus or any other fluid into the lungs | | | | | |
|  | Accidental Injury | - Minor external bleeding or injury, | - Need of minor surgery | - Fracture OR - Need of major surgery OR - Major bleeding OR - Significant intracranial bleeding | - Accompanied by cardiorespiratory compromise | - Death |
|  | Definition: A disorder characterized by injury to body due to accident like fall or violent contact with external object, including accidental asphyxia | | | | | |
|  | BPD | - Need of oxygen or respiratory support for at least 28 days but free of oxygen at 56 days/36 weeks PMA (mild BPD) | - Need of oxygen or respiratory support for at least 28 days but need of oxygen less than 30% at 56 days/36 weeks PMA (moderate BPD) | - Need of oxygen or respiratory support for at least 28 days but need of oxygen more than 30% or positive respiratory pressure at 56 days/36 weeks PMA (severe BPD) | - Severe BPD with frequent BPD spells, OR persistent desaturations - Presence of cor pulmonale | - Death |
|  | Definition: A disorder characterized by prolonged (>28 days) requirement of oxygen therapy or respiratory support, respiratory distress and X-ray picture showing diffuse haziness or areas of hyperinflation and collapse/fibrosis | | | | | |
|  | Coagulopathy/DIC |  | - Laboratory evidence but no significant clinical bleeding | - Significant clinical bleeding along with laboratory evidence |  | - Death |
|  | Definition: A disorder characterized by state if altered coagulation status due to abnormal activation of coagulation cascade | | | | | |
|  | Cholestasis/hepatitis |  | - Asymptomatic cholestasis (TSB<12 mg/dL) | - Cholestasis associated with complications like significant bleeding, encephalopathy , impairing growth OR - TSB 12 mg/dL or more | - Cholestasis associated with hepatic failure | - Death |
|  | Definition: A disorder characterized by inflammation of hepatic parenchyma and/or biliary tract which manifests as raised blood levels of liver enzymes and serum bilirubin | | | | | |
|  | Extravasation injury | - skin erythema and small swelling of extremity | - Large swelling, OR - Superficial skin necrosis | - Deep skin necrosis |  |  |
|  | Definition: A disorder characterized by injury caused to skin due to extravasation of intravenous fluid | | | | | |
|  | Encephalopathy |  | - Transient (lasting less than 72 h) and mild abnormality in gestation-appropriate muscle tone, consciousness and reflexes, OR - HIE stage 1 | - Prolonged (72 h or more) OR - Moderate to severe, OR - HIE stage 2 abnormalities in gestation-appropriate muscle tone, consciousness and reflexes OR - Presence of seizures | - Encephalopathy causing cardiorespiratory compromise resulting in need of intubation or inotropic support, OR - HIE stage 3 | Death |
|  | Definition: A disorder characterized by abnormality in muscle tone, consciousness and reflexes | | | | | |
|  | Gastroenteritis | - Gastroenteritis without dehydration | - Gastroenteritis with moderate dehydration | - Gastroenteritis with severe dehydration | - Gastroenteritis with shock | - Death |
|  | Definition: A disorder characterized by inflammation of gastrointestinal tract manifesting as loose motions, vomiting, fever and dehydration. If gastroenteritis is a manifestation of sepsis- include it under heading of sepsis not under gastroenteritis heading. | | | | | |
|  | Gangrene |  | - Gangrene of distal phalanx | - Gangrene extending >1 phalanx of any digit or metacarpals/metatarsals | - Gangrene including long bones of limbs |  |
|  | Definition: A disorder characterized by gangrene of a part of body.  Note: If gangrene is caused due to sepsis, then sepsis may only be reported as SAE.  However, if gangrene is caused due to sampling, in such cases, gangrene may be reported as SAE. | | | | | |
|  | Hypoglycemia | - Blood glucose 20-40 single episode, asymptomatic, treated with oral feeds | - More than one episode of blood glucose 20-40 mg/dL OR - Blood glucose <20 OR - Symptoms other than seizures OR - Need for glucose infusion up to 12 mg/kg/min | - Seizures OR - Need of intravenous glucose infusion @ >12 mg/kg/min or persisting for > 7 days |  |  |
|  | Definition: A disorder characterized by blood glucose concentration less than 40 mg/dL | | | | | |
|  | Hyperglycemia | - NOT needing treatment with insulin | - Need of treatment with insulin |  |  |  |
|  | Definition: A disorder characterized by blood glucose concentration greater than 150 mg/dL | | | | | |
|  | Hypothermia | - axillary temperature 36.0^o^C-36.4^o^C | - axillary temperature 32.0^o^C-35.9^o^C | - axillary temperature <32.0^o^C |  |  |
|  | Definition: A disorder characterized by axillary temperature less than 36.5^o^C | | | | | |
|  | Hyperthermia | - axillary temperature 37.6^0^C-38.0^o^C | - axillary temperature 38.1^o^C-40.0^o^C | - axillary temperature >40.0^o^C |  |  |
|  | Definition: A disorder characterized by axillary temperature more than 37.5^o^C | | | | | |
|  | Hypernatremia | - Serum sodium 146-150 mEq/L | - Serum sodium 151-160 mEq/L | - Serum sodium 161-170 mEq/L | - Serum sodium >170 mEq/L OR - One accompanied by clinical features of seizures or altered consciousness | - Death |
|  | Definition: A disorder characterized by increase in concentration of sodium ion in blood | | | | | |
|  | Hyperkalemia |  | - Serum potassium 5.5-6.5 mEq/L | - Serum potassium 6.5-8.0 mEq/L | - Serum potassium >8 mEq/L | - Death |
|  | Definition: A disorder characterized by increase in concentration of potassium ion in blood | | | | | |
|  | Hyponatremia | - Serum sodium 130-134 mEq/L | - Serum sodium 120-129 mEq/L | - Serum sodium 110-119 mEq/L | - Serum sodium <110 mEq/L OR - One accompanied by clinical features of seizures or altered consciousness | - Death |
|  | Definition: A disorder characterized by decrease in concentration of sodium ion in blood | | | | | |
|  | Hyperbilirubinemia | - Hyperbilirubinemia without need of therapy | - Hyperbilirubinemia needing treatment with phototherapy | - Hyperbilirubinemia needing treatment with blood exchange transfusion | - Hyperbilirubinemia with acute bilirubin encephalopathy | - Death |
|  | Definition: A disorder characterized by increase in indirect bilirubin levels in blood | | | | | |
|  | Inguinal hernia |  | - Reducible hernia, unobstructed | - Obstructed | - Strangulated |  |
|  | Definition: A disorder characterized by herniation of abdominal contents in to inguinal canal | | | | | |
|  | Lung collapse |  | - Lung collapse involving segments/lobe NOT causing new symptoms, or resulting in increase in oxygen/ventilatory requirement | - Lung collapse with increased oxygen or ventilation requirements | - Lung collapse resulting in hypoxia or hypercarbia needing high frequency ventilation | - Death |
|  | Definition: A disorder characterized by collapse/atelectasis of lung or its segment/lobe | | | | | |
|  | Nasal trauma | - Self-healing nasal trauma NOT causing permanent disfigurement |  | - Nasal trauma which causes severe and permanent disfigurement OR - One needing surgical correction | -- | -- |
|  | Definition: A disorder characterized by trauma to nose due to respiratory support (CPAP) | | | | | |
|  | Neutropenia | - Asymptomatic and NOT associated with systemic infection, NOT needing any therapeutic intervention | - Asymptomatic and NOT associated with systemic infection, but needing therapeutic intervention | - Associated with systemic infection |  |  |
|  | Definition: A disorder characterized by decrease (below gestation and postnatal age specific threshold) in number of neutrophils in peripheral blood film | | | | | |
|  | NEC | - | - NEC stage 1 as per Walsh Kleigmann modification of Bell’s classification | - NEC stage 2 as per Walsh Kleigmann modification of Bell’s classification | - NEC stage 3 as per Walsh Kleigmann modification of Bell’s classification | - Death |
|  | Definition: A disorder characterized by inflammation of gut which may progress to intestinal necrosis | | | | | |
|  | Osteopenia | - Asymptomatic, diagnosed on imaging studies | - Causing fracture or prolongation of respiratory support |  |  |  |
|  | Definition: A disorder characterized by decreased bone mass | | | | | |
|  | Pressure sore |  | - Small (<2 cm)- healing happens in <1 wk | - Major pressure sore (>2 cm) and requiring surgical intervention or non-healing pressure sore |  |  |
|  | Definition: A disorder characterized by formation of ulcers over pressure sites | | | | | |
|  | Polycythemia | - Asymptomatic NOT needing treatment | - Symptomatic needing treatment with fluid relaxation or partial exchange transfusion | - Associated with complications such as NEC, thrombosis |  |  |
|  | Definition: A disorder characterized by increased hemoglobin or packed cell volume in blood as per gestation and postnatal gestation norms | | | | | |
|  | Pleural effusion |  | - Minimal fluid in the pleural cavity requiring no/ minimal change in respiratory support | - Free fluid in the pleural cavity requiring significant increase in respiratory support or requiring ICD drainage | - Fluid in pleural cavity resulting in circulatory collapse | - Death |
|  | Definition: A disorder characterized by presence of free fluid in pleural cavity | | | | | |
|  | PPHN/PAH |  | - Clinically insignificant PPHN requiring no/minimal increase in respiratory support | - Clinically significant PPHN needing significant increase in respiratory support | - Clinically significant PPHN needing high frequency ventilation or ECMO or iNO | - Death |
|  | Definition: A disorder characterized by increased pulmonary vascular pressure resulting in right to left shunting of blood diagnosed by echocardiography | | | | | |
|  | Peritonitis |  |  | - Peritonitis unaccompanied by perforation of gut | - Peritonitis accompanied by perforation of gut | - Death |
|  | Definition: A disorder characterized by inflammation of peritoneal membranes. | | | | | |
|  | PDA | - Asymptomatic PDA | - PDA needing medical treatment | - PDA needing surgical treatment or resulting in cardiac failure | - PDA causing life threatening complications such as AKI, severe pulmonary hemorrhage, NEC stage 3 | - Death |
|  | Definition: A disorder characterized by persistent non-closure of ductus arteriosus | | | | | |
|  | PVL | - Periventricular flare without cyst formation | - Periventricular leuokomalacia with cysts formation in frontal, parietal regions | - Periventricular leuokomalacia with cysts formation in occipital periventricular white matter | - | - |
|  | Definition: A disorder characterized by hypoxic-ischemic injury to periventricular brain resulting in disability | | | | | |
|  | Periventricular- intraventricular haemorrhage (P/IVH) | - Grade 1 IVH | - Grade 2 IVH | - Grade 3 IVH, OR - PVHI, OR - Any grade IVH manifesting as seizure or post-hemorrhagic hydrocephalus | - Post-hemorrhagic hydrocephalus needing shunt placement, OR - IVH causing significant respiratory compromise requiring ventilation or hemodynamic compromise requiring vasopressors | - Death |
|  | Definition: A disorder characterized by bleeding into periventricular- intraventricular space in brain | | | | | |
|  | Pulmonary hemorrhage | - Occasional small bleed occurring spontaneously or during endotracheal suction requiring no/minimal change in respiratory support | - Recurrent small bleeds, does not require a transfusion/significant hike in respiratory support. | - Need for blood/component transfusion, OR - Need for significant augmentation of respiratory support, OR - Prolongation of ventilation | - Shock requiring vasopressors | - Death |
|  | Definition: A disorder characterized by bleeding from the bronchial wall and/or lung parenchyma manifested as endotracheal bleeding or bleeding from mouth in a non-intubated baby. | | | | | |
|  | ROP | - Any ROP which is less severe than type 1/2 (this does not refer to stage 1 or 2 ) ROP. | - Type 2 ROP as described in ETROP study | - Type 1 ROP as described in ETROP study or ROP needing treatment | -- | -- |
|  | Definition: A disorder characterized by abnormal fibrovascular proliferation in retina  **Type 1 ROP** - any of 3: (1) Zone I, any stage ROP with plus disease, (2) Zone I, stage 3 ROP without plus disease, or (3) Zone II, stage 2 or 3 ROP with plus disease  **Type 2 ROP**- any of two: (1) zone I, stage 1 or 2 ROP without plus disease, (2) Zone II, stage 3 ROP without plus disease | | | | | |
|  | Seizures |  | - Single episode of seizures without cardiorespiratory compromise due to transient metabolic abnormalities like hypoglycaemia, hypocalcaemia | - Multiple episodes of seizures OR - Seizure needing treatment with antiepileptic drug | - Status epilepticus OR - Seizures causing cardiorespiratory compromise | - Death |
|  | Definition: A disorder characterized by abnormal involuntary movements of muscles | | | | | |
|  | Spontaneous Intestinal Perforation (SIP) |  |  | - SIP NOT needing surgical management by laparotomy and NOT causing cardiorespiratory compromise | - SIP needing surgical management by laparotomy or leading to cardiorespiratory compromise | - Death |
|  | Definition: A disorder characterized by perforation of intestine without any clinical or intraoperative or pathological evidence of NEC | | | | | |
|  | Shock |  | - Hypotension or decreased organ perfusion lasting for <6 hours needing volume expander and resolving without consequence | - Hypotension or decreased organ perfusion needing /vasopressor(s)/ ionotropes | - Catecholamine resistant shock | - Death |
|  | Definition: A disorder characterized by inability of circulatory system to meet metabolic requirement of tissues | | | | | |
|  | Subglottic stenosis | - Transient (<24 hours) increase in oxygen requirement, respiratory difficulty or stridor or needing non-invasive respiratory support or need of inhalation drug therapy | - Subglottic stenosis resulting in extubation failure, OR - Need of systemic steroid therapy or tracheostomy | - Significant respiratory distress resulting in more than 2 extubation failures or need for tracheostomy |  |  |
|  | Definition: A disorder characterized by stenosis in the subglottic airway | | | | | |
|  | Sepsis (irrespective of etiology- bacterial, viral, fungal, protozoal) includes pneumonia, meningitis or bone-joint infection` | - | - Culture negative sepsis, OR - Ventilator associated pneumonia requiring no/minimal increase in respiratory support | - Culture positive sepsis OR - Meningitis OR - Encephalitis, OR - Presence of sclerema, OR - Ventilator associated pneumonia requiring significant increase in respiratory support | - Septic shock requiring vasopressors, OR - Multi-organ dysfunction such as presence of AKI, DIC-bleeding, OR - Ventilator associated pneumonia resulting in persistent desaturations despite high level ventilator support | - Death |
|  | Definition: A disorder characterized by the presence of pathogenic microorganisms in the bloodstream that cause a rapidly progressing systemic reaction that may lead to shock and/or end organ failure | | | | | |
|  | Thromboembolism | - Asymptomatic, NOT needing treatment | - Causing gangrene of distal phalanx | - Causing gangrene extending >1 phalanx of any digit or metacarpals/metatarsals | - Causing gangrene including long bones of limbs or any internal organs like brain, kidneys or lungs |  |
|  | Definition: A disorder characterized by thromboembolic phenomenon involving a vascular system | | | | | |
|  | Thrombocytopenia | - NOT associated with bleeding and above the threshold for platelet transfusion | - NOT associated with active bleeding but needing platelet transfusion | - Associated with bleeding or platelet count less than 20,000 | - Associated with intracranial bleeding | - Death |
|  | Definition: A disorder characterized by decrease in number of platelets (less than 150,000/µL) in peripheral blood | | | | | |

**S2: Guidance document for deciding compensation for trial-related injury in GLSE trial**

| Title of the Project | **Evaluating the efficacy and safety of an innovative and affordable Goat Lung Surfactant for the treatment of respiratory distress syndrome in preterm neonates: a multi-site randomized clinical trial** |
| --- | --- |
| Sponsor | Director, AIIMS, New Delhi |
| PI | Dr Ramesh Agarwal |
| Partner | Clinical Development Service Agency, Faridabad |
| Number of sites | 12 |
| Date of the Meeting | 29^th^September, 2015 |

# **Background**

# Prematurity- a leading cause for neonatal deaths. Preterm births account for 27% of 3.1 million neonatal deaths occurring globally every year. Millennium Development Goal-4 (MDG-4) stipulates a reduction of two-thirds in deaths in children aged less than 5 years.^1^

# This could be accomplished only by achieving a substantial reduction in neonatal deaths including those related to preterm births.

LMICs contribute 90% of the burden of neonatal deaths with India being the largest contributor as a country (GAPPS 2012). *It is estimated that approximately half of prematurity related deaths are due to respiratory distress syndrome (RDS) in view of* unavailability of proven treatment modalities including surfactant.RDS in preterm neonates occurs due to the deficiency of lung surfactant resulting in impaired oxygenation and breathing difficulty. In general, the more premature the infant, less is the surfactant production and higher the probability of RDS.

Exogenous surfactant is obtained from animal lungs by either lung lavage or mincing of lung tissue of slaughtered animals like pork and calves (natural surfactant). Treatment of preterm babies with RDS with exogenous surfactant obtained from natural sources such as bovine or porcine (surfactant replacement therapy, SRT) reduces mortality, pneumothorax, sepsis and other important morbidities. SRT has saved enumerable lives across the world since its introduction in 1990s. ^2^

However, despite availability of exogenous surfactant as imported products, majority of neonates needing SRT in India (90% of 300 000 every year) do not get it due to high cost (Rs 8000 to 15000 per dose).^3^ Government of India and World Health Organization have, therefore, identified development of affordable exogenous surfactant as an important priority.

# **Development of an affordable surfactant**

AIIMS team developed goat lung surfactant extract (GLSE) and demonstrated the proof of concept. Subsequent product development was carried out by Cadila Pharmaceuticals, Ahmadabad, which got marketing permission from FDCA, Gujarat (Cadisurf). However, GLSE has not been used in humans till now due to lack of randomized trials in human subjects.

# **The proposed trial**

AIIMS, New Delhi plans to conduct a randomized trial to test the non-inferiority of GLSE with a standard product (Survanta, Abbvie; formerly Abbott, USA). This is an **investigator driven trial** with funding support from Wellcome Trust, UK. Cadila Pharmaceuticals Limited would provide trial batch of Cadisurf and would not have any role in design, execution, analysis and interpretation of data and reporting of the results of the trial, which remain total prerogative of investigators.

If GLSE is found to be non-inferior to Survanta, the former would be available at a reduced price (1/4 to 1/5 of current cost; approximately Rs 2000). This would make an affordable surfactant available for India and other LMICs and save millions of lives in years to come.

# **Ascertainment of trial related injury**

As per Drugs and Cosmetic Rules, if it is a CDSCO regulated trial, sponsor has to provide compensation to the trial participants in case there is a trial related injury (death or other serious adverse event; SAE). In addition, the participants have to be provided necessary treatment free of cost until the resolution of SAE or it is proved that SAE is unrelated to the trial. CDSCO has provided detailed guidelines with respect to calculation of quantum of compensation in different situations.^4^

# **Challenges in ascertainment of trial related injury in GLSE trial**

Preterm neonates (26 to 32 weeks gestation with respiratory distress syndrome requiring surfactant replacement therapy) proposed to be enrolled in this (GLSE) trial carry high risk of mortality despite providing standard of care in level-3 neonatal units. Apart from RDS, these neonates suffer from other life threatening morbidities.

There are two important challenges in ascertaining relatedness of SAE and deciding the quantum of compensation in these neonates

1. The current CDSCO guidelines recommend calculation of compensation amount based on the (a) risk factor assigned as per expected survival of the study participants at the time of enrolment (b) age of the participant (c) a base amount which is Rs 800,000 at present. However, it is difficult to decide the risk factor in GLSE trial for two reasons (i) Despite providing standard of care, one-third to one half of neonates die due to serious underlying disease, and (ii) the majority of these deaths occur within hours to days, while surviving neonates have near normal life expectancy.
2. These neonates have significant co-morbidities as a result of underlying prematurity apart from RDS. Some of these co-morbidities can happen as a result of SRT. For a given SAE, it becomes difficult to decide if it is a consequence of the underlying disease process or a complication of SRT.

**Issue 1: Which risk category should be assigned to the preterm neonates having eligibility criteria for GLSE trial for calculation of compensation amount?**

To answer this question, the Committee examined following data in preterm neonates of 26 to 32 weeks gestation and suffering from RDS in Indian setting:

1. **Mortality rate when the neonates were not treated with SRT** Mortality rate for preterm neonates suffering from respiratory distress syndrome (RDS) who were not given SRT (as the surfactant was unavailable/unaffordable in India; late 90s to early 2000s) is 60% – 70% (Sources: Data from AIIMS, PGI and NNPD 2002-03).
2. **Mortality rate when neonates were treated with SRT**

Even providing SRT and other standard of care, the mortality rate was 30% to 50% in preterm neonates at the centres that will be participating in GLSE trial (Sources: data obtained from unit statistics of the participating sites caring inborn babies. We did not include outborn babies as the denominator data is not available).

1. **Time to death**

AIIMS data showed that median (range) time to death (n=56) was 12 days (0-290) days. Over 90% of mortality occurred within 180 days of birth**.**

1. **Co-morbidities in such infants**

AIIMS data showed that approximately one third of these infants suffer from one or more other life threatening conditions.

The Sponsor, the Site Investigators and site IECs should take the **Risk Factor** of ‘0.5’ as in case of terminally ill patients for calculation. They need to submit their recommendation to licensing authority (CDSCO) for quantum of compensation. CDSCO makes the final decision regarding the quantum of compensation as per Rule 122DAB and Appendix XII to Schedule Y of the Drugs and Cosmetics Rules.

# **Issue 2: What should be the framework of assigning a death or other serious adverse event as trial related injury?**

Under Rule 122 DAB, sub-rule 5 (Notification No. GSR53E dated: 30.01.2013) states that: ‘The Sponsor or its representative, whosoever had obtained permission from the Licensing Authority (CDSCO) for conduct of the clinical trial, shall provide financial compensation, if the injury or death has occurred because of any of the following reasons, namely:-

1. Adverse effect of investigational product(s)
2. Any clinical trial procedures involved in the study
3. Violation of the approved protocol, scientific misconduct or negligence by the sponsor or his representative or the investigator
4. Failure of investigational product to provide intended therapeutic effect (where the standard care, though available, was not provided to the subject as per the clinical trial protocol)
5. Use of placebo in a placebo-controlled trial
6. Adverse effects due to concomitant medication excluding standard care, necessitated as part of approved protocol
7. Injury to the child in-utero because of the participation of parent in clinical trial.

In general, payment of compensation in case of SAE occurring with standard of care arm (Survanta arm in this trial) is NOT required. However, compensation has to be provided if there is any violation of the approved protocol, scientific misconduct or negligence by the sponsor or his representative or the Investigator even in the “standard of care” arm.

The Licensing Authority (CDSCO) will finally decide the relatedness of the SAE to the drug or the clinical trial procedures. In case it is determined to be related to the trial procedures or as per the laid down criteria under Rule 122DAB, compensation becomes applicable. This implies that compensation must be paid as per the orders of the Licensing Authority**. In addition, the sponsor has to provide free medical management to patient for any adverse event as per Drugs and Cosmetic Rules.**

Preterm neonates of (26 to 32 weeks of gestation) suffer from a variety of serious illnesses such as RDS, pneumothorax, patent ductus arteriosus (PDA), necrotizing enterocolitis (NEC), retinopathy of prematurity (ROP), intraventricular hemorrhage (IVH), periventricular leuocolamacia (PVL), sepsis, pulmonary hemorrhage and bronchopulmonary dysplasia. SRT using animal derived surfactant has shown to significantly reduce most of these morbidities.

SRT is safe modality of care, which is generally associated with transient adverse events such as transient desaturations, pallor, bradycardia, and hypotension, which gets corrected by temporarily stopping the procedure and ensuring good ventilation. In addition, there may be reflux of surfactant in endotracheal tube or tube may be blocked. The SRT using synthetic surfactant (used in past; now sparingly used) compared to no surfactant was associated with increased incidence of apnea of prematurity and non-significant increase in pulmonary hemorrhage.

To evaluate the possible association of SAE and SRT, the committee examined following data:

1. **Incidence of SAE and death when preterm neonates with RDS are treated with animal derived surfactant compared to placebo or no treatment**

Since, the SRT has become standard of care in past 25 years; there are no recent data from randomized trial comparing SAEs in neonates receiving SRT versus those who did not. We therefore resorted to older studies carried out before SRT became the standard of care. A Cochrane review (updated in 2009) compared animal derived surfactant extract (natural surfactant) for treatment of RDS versus no surfactant. This review included 13 trials and 1605 neonates. All the studies were conducted in high income country settings. The review showed that SRT compared to placebo reduced the risk of mortality (by 9%), pneumothorax (by 17%), PIE (by 20%), air leak (by 16%) and BPD or death at 28 days of life (by 11%). There is no effect on incidence of pulmonary hemorrhage, PDA, NEC, Sepsis, BPD or ROP.

1. **Incidence of SAE and death when preterm neonates at high risk of RDS are treated with prophylactic SRT versus rescue (directed) SRT**

SRT can be given to all neonates who at higher risk of developing RDS (such as neonates <28 weeks gestation. Some of these neonates will not suffer from RDS later. Therefore prophylactic SRT compared to rescue SRT results in much liberal use of surfactant) or it can be given only when RDS actually sets in (rescue therapy resulting in restricted use). One can get some idea of excess risk due to SRT by comparing event rates of SAE in prophylactic versus rescue groups. A Cochrane systematic review (updated in 2012) compared prophylactic versus rescue SRT. The review included 11 trials and 4507 neonates. All the studies were conducted in high income country settings.

Prophylactic SRT (liberal use) as compared to rescue therapy (restricted use) results in reduction of mortality (by 1%), pneumothorax (by 1%), PIE (by 2%) and BPD or death by 28 days (by 4%). There was no significant effect on BPD, air leak, pulmonary hemorrhage, PDA, Sepsis, NEC or ROP.

1. **Experience in LMICs**

There no randomized trial from India and other low and middle income countries (LMIC) examining the efficacy and safety of surfactant. As the SRT had already become standard of care in developed nations, LMICs just adopted it once surfactant became available to them.

***India***

Experience at AIIMS, New Delhi showed that, upon introduction of surfactant use in clinical practice in around year 2000, the mortality in the NICU babies reduced in extremely low birth preterm neonates (birth weight <1000 gm) (59 [42%] in 2010-13 vs. 70 [51%] in 1994-2000) and neonates between 1000 and 1049 gm (22 [7.6%] vs. 16 [11.8%]).

In a before-and-after study from PGI, Chandigarh, neonates with RDS who received surfactant (n=88) compared to those who did not (n=119) had lesser incidence of mortality (33, 37.5% vs. 67, 56.3%), air leak (7; 7.9% vs. 11, 9.9%), BPD at 28 days 8/65 (10.9%) vs. 12/48 (25.0%), PDA (41; 46.6% vs. 70; 58.8%) and sepsis (28; 31.8% vs. 60; 50.4%).

***Other LMICs***

In retrospective studies comparing surfactant treated babies with historical control from South Africa (2 studies), Latin American countries (1 study), Malaysia (5 studies), and China (4 studies) showed that introduction of surfactant resulted in significant reduction in neonatal mortality.

1. **Pre-clinical toxicity studies of Cadisurf**

*Toxicity studies*

Acute Toxicity studies conducted by intraperitoneal and intratracheal administration of GLSE in rats, mice, rabbits and guinea pigs have demonstrated no adverse events with a maximal single dose of 2000 mg/kg (20 times the clinically recommended dose)

**Summary of decisions For GLSE trial to be conducted at 12 sites in India with AIIMS being sponsor and Dr Ramesh Agarwal being the PI:**

1. The risk factor to be taken as 0.5 (terminally sick) for calculation of compensation for trial related injury.

2. The trial participants will have high rate of many serious morbidities. Some of them may be related to trial drug/procedures. It is advisable to use a formal tool namely WHO scale or Hybrid scale (developed by AIIMS IEC) for assigning relatedness of SAE. It is important that the Sponsor, Investigators and the IECs give their opinion in following terms:

a. If SAE was RELATED OR UNRELATED to trial drug/procedure

b. Adequate justification and documents for their decision in regard to relatedness

3. CDSCO finally decides if the given SAE is related or unrelated to trial drug/procedure, and the quantum of compensation based on the reports received from investigator, sponsor and site IEC.

**Serious Adverse Event (SAE) and Reporting Timelines**

1. **Serious Adverse Event:** An adverse event or suspected adverse reaction is considered "serious" if, in the view of either the investigator or sponsor, it results in **any of the following**:
   - Death
   - A life-threatening adverse event
   - Inpatient hospitalization or prolongation of existing hospitalization
   - A persistent or significant incapacity or disability or substantial disruption of the ability to conduct normal life functions, or
   - A congenital anomaly/birth defect
2. **SAE Reporting Timelines**

Any Unexpected Adverse Events or Serious Adverse Events including death due to any cause, which occur to any subject entered into study treatment in this study, whether or not related to the investigational product must be reported:

- The investigator will report the SAE within 24 hours of occurrence of SAE (regulatory requirement). The SAE will be reported written (SAE Form, and SAE narrative as per schedule Y, attached, Annex 10 & 11) by fax, email to Licensing authority (CDSCO), AIIMS (the sponsor), CDSA (sponsor representative), and the Chairman of Institutional Ethics committee (IEC).
- The investigator will send a detailed report (SAE Form and SAE narrative as per schedule Y, attached, Annex 10 & 11) within 7 calendar days of occurrence of any SAE (death or other than death) to AIIMS and CDSA.
- The investigator will send a detailed report within 14 calendar days of occurrence of any SAE (death or other than death) (SAE narrative as per schedule Y, attached, Annex 11) to Licensing Authority (CDSCO), IEC and Head of the institution where the trial has been conducted (regulatory requirement).
- AIIMS to forward report of SAE (death and other than death) after due analysis (SAE narrative as per schedule Y, attached, Annex 11) to the Chairman of the IEC, Licensing authority and Head of the institution within 14 days of occurrence of the event (regulatory requirement).
- In case of SAE  (death and other than death), IEC shall forward its report after due analysis within 30 calendar days of occurrence of SAE along with opinion on financial compensation, if any, to be paid by AIIMS (regulatory requirement)
- In case of SAE of death, the Licensing authority will send all the reports to the Chairman of Expert Committee for recommendations on compensation. The Chairman of Expert Committee will send the recommendations for compensation within 105 days after determining the cause of injury or death (regulatory requirement).
- The Licensing authority will give its order on compensation within 150 calendar days of occurrence of SAE (death or other than death) to the sponsor.
- The Sponsor will pay the compensation within 30 calendar days after receiving the order from Licensing authority.
- In case the investigator fails to report any SAE within stipulated period, she/he shall furnish the reason for the delay to the satisfaction of Licensing Authority along with report of SAE (regulatory requirement).
- The Investigator will be requested to submit a report. Wherever applicable, information from relevant hospital records and autopsy reports should be obtained.
- The immediate and follow-up reports are to identify the subject by the unique subject number and initials, and not by the subject's name or address.
- Investigators should follow-up subjects with SAEs occurring at any time following study medication administration until the event has disappeared or until the condition has stabilized.

**Formula to determine the quantum of compensation in case of clinical trial related injury or death**

The Drugs and Cosmetics Rules have been amended vide GSR 53 (E) dated 30 – 01 - 2013 inserting a Rule 122DAB and a new Appendix - XII in Schedule “Y‟. The amendment specifies the procedure for processing of reports of Serious Adverse Events (SAEs) including deaths occurring during clinical trial to arrive at the cause of death/injury to the subject, and to determine the quantum of compensation, if any, to be paid by the Sponsor or his representative, whosoever have obtained permission from the Drugs Controller General (India) {DCG (I)} in a time bound manner.

NOTE: However, in case of patients whose expected mortality is 90% or more within 30 days, a fixed amount of Rs. 2 lacs should be given

Quantum of Compensation:

1. **Trial related SAE (Serious Adverse Event) of Death**

| Compensation = (B x F x R) / 99.37 |
| --- |

- B = base amount i.e., Eight Lacs
- F = 228.54
- R = Risk Factor. 0.5 for the present study

Compensation = (8X228.54X0.5)/ 99.37

~ 9.12 lakh

1. **Trial related SAE causing Permanent Disability to the Subject**

| Compensation = (D x 90 x C) / 100*100 |
| --- |

D= % of Disability

C= Quantum of Death compensation as calculated above

Examples:

1. For 25% disability

Compensation = 25X90X9.12/100X100

= 2.05 lakh

1. For 50% disability

Compensation = 50X90X9.12/100X100

= 4.10 lakh

1. For 100% disability

Compensation = 100X 90X 9.12/100X100

= 8.21 lakh

1. **Trial related SAE causing Congenital Anomaly or Birth Defect**

Quantum of compensation will be calculated with half base amount ie., 4 lacs

| Compensation = {(B/2) x F x R} / 99.37 |
| --- |

1. **Trial related SAE causing Life-threatening Disease**

| Compensation = 2*N*W |
| --- |

N= No of days the subject under life threatening Situation requiring medical care, irrespective of number of days of hospitalization

W= Minimum Wages per day of Unskilled worker (in Delhi)

Example:

If N= 60 days

Compensation = 2X60X353^*^

= Rs 42360

*Rates from Order by Government of NCT of Delhi F.No.12(142)/13/mw/Lab/2231 dated 16.10.2015

1. **Trial related Reversible Resolved SAE**

| Compensation = 2*N*W |
| --- |

N= No of days of hospitalization

W= Minimum Wages per day of Unskilled worker

Example:

If N= 10 days

Compensation = 2X10X353^*^

= Rs 7060

* Rates from Order by Government of NCT of Delhi F.No.12(142)/13/mw/Lab/2231 dated 16.10.2015

**AIIMS Hybrid tool for assessing causality of SAE**

| **Criteria** | **Response** |
| --- | --- |
| Temporal relationship (Yes/No) | Yes |
| Expectedness of investigational drug (Yes/No) | Yes |
| Class effect (Yes/No) | No |
| Disease progression possibility (Yes/No) | Yes |
| Other possible implicating cause (Yes/No) | Yes |
| Similar SAE reported in literature (Yes/No) | Yes |
| After stoppage of drug or dose reduction, SAE intensity decreased? (Not applicable/Yes/No/No information) | Yes |
| After re-exposure of drug, SAE intensity increased? (Not applicable/Yes/No/No information) | No information |
| Compensation required (Yes/No) | No |
| Recommended Compensation amount | No |

**FORMAT FOR REORTING SAE**

| **1. REPORT DETAILS** |
| --- |
| **Report type** (tick one): Initial  Follow-up |

| **2. PARTICIPANT DETAILS** | | |
| --- | --- | --- |
| **Initials:** | **DOB** (dd/mm/yyyy): | **Sex:**  Male  Female |

| **3. EVENT DETAILS** | |
| --- | --- |
| **Date of onset** (dd/mm/yyyy): | **Diagnosis:** |
| **Description of SAE in medical terms:** | |
| **Seriousness Criteria** (check all that are relevant to the event):  Participant died  Inpatient hospitalisation or prolongation of existing inpatient hospitalisation  Life-threatening  Involved persistent or significant disability or incapacity  Congenital anomaly/  Other significant medical events (as defined in protocol)  birth defect | |
| **Severity of event:**  Mild  Moderate  Severe | |

| **4. STUDY TREATMENT**  Check box if medication stopped | | | | | | | | |
| --- | --- | --- | --- | --- | --- | --- | --- | --- |
| **Investigational**  **Product (IP)** | **Dose**  **/schedule** | **Route of administration** | **Start date**  (dd/mm/yyyy) | **End date**  (dd/mm/yyyy) | **Causally Related to IP?**  ***Tick appropriate Box*** | | | |
|  |  |  |  |  | **Unrelated** | **Possibly Related** | **Probably Related** | **Definitly related** |
| 1. |  |  |  |  |  |  |  |  |
| 2. |  |  |  |  |  |  |  |  |
| 3. |  |  |  |  |  |  |  |  |
| 4. |  |  |  |  |  |  |  |  |

| **5. CONCOMITANT DRUGS RELEVANT TO THE SAE***(do not include therapy used to treat the SAE)*  Check box if no relevant concomitant medication | | | | | | |
| --- | --- | --- | --- | --- | --- | --- |
| **Drug name** | **Dose/schedule** | **Route of administration** | **Reason for use** | **Start date**  (dd/mm/yyyy) | **End date**  (dd/mm/yyyy) | **Continued?**  (Y/N) |
| 1. |  |  |  |  |  |  |
| 2. |  |  |  |  |  |  |
| 3. |  |  |  |  |  |  |
| 4. |  |  |  |  |  |  |
| 5. |  |  |  |  |  |  |

| **6. MEDICATION GIVEN TO TREAT THE SAE**  Check box if no relevant medication | | | | | | |
| --- | --- | --- | --- | --- | --- | --- |
| **Drug name** | **Dose/schedule** | **Route of administration** | **Reason for use** | **Start date**  (dd/mm/yyyy) | **End date**  (dd/mm/yyyy) | **Continued?**  (Y/N) |
| 1. |  |  |  |  |  |  |
| 2. |  |  |  |  |  |  |
| 3. |  |  |  |  |  |  |
| 4. |  |  |  |  |  |  |
| 5. |  |  |  |  |  |  |
| 6. |  |  |  |  |  |  |
| 7. |  |  |  |  |  |  |
| 8. |  |  |  |  |  |  |
| 9. |  |  |  |  |  |  |
| 10. |  |  |  |  |  |  |
| 11. |  |  |  |  |  |  |

| **7. MEDICAL HISTORY (list relevant medical history):**  Check box if no relevant medical history | | | | |
| --- | --- | --- | --- | --- |
| **Condition** | **Start Date**  (dd/mm/yyyy) | **End date**  (dd/mm/yyyy) | **Ongoing**  **(Y/N)** | **Medication required**  Y/N |
| 1. |  |  |  |  |
| 2. |  |  |  |  |
| 3. |  |  |  |  |
| 4. |  |  |  |  |

| **8. RELEVANT TEST/LABORATORY FINDINGS***(include only the results relevant to the SAE diagnosis or course of SAE)* | | | | | |
| --- | --- | --- | --- | --- | --- |
| **Test/lab finding** | | **Date**  (dd/mm/yyyy) | **Value** | | **Units** |
| 1. | |  |  | |  |
| 2. | |  |  | |  |
| 3. | |  |  | |  |
| 4. | |  |  | |  |
| 5. | |  |  | |  |
| 6. | |  |  | |  |
| 7. | |  |  | |  |
| Comment on test/laboratory findings (if none, mark as NA) | | | | | |
| **9. ACTION TAKEN (check all that are relevant to the SAE)** | | | | | |
| No action taken | IP permanently discontinued due to this SAE  *If multiple IMPs used, please record which IMP(s) have been discontinued:* | | | Concomitant medication taken | |
| IP dose adjusted/temporarily interrupted  *If multiple IMPs used, please record which IMP(s) have been adjusted/interrupted:* | Non-drug therapy given | | | Hospitalisation/prolonged hospitalisation | |
| **10. OUTCOME OF SAE** | | | | | |
| Completely recovered  Date of recovery (dd/mm/yyyy): | Condition still present and unchanged | | | Recovered with sequelae Date (dd/mm/yyyy): | |
| Condition deteriorated | Condition improving | | | Death  Date of death (dd/mm/yyyy)  Post mortem? Yes  No  *(If YES, Please attach post-mortem report with this form)* | |

| **11. ADDITIONAL INFORMATION** |
| --- |
|  |

| **12. INFORMATION SOURCE** | |
| --- | --- |
| Name, address and telephone number of PI: |  |
| Date of report (dd/mm/yyyy): |  |
| PI signature: |  |
| **ALL REPORTS MUST BE SIGNED AND DATED BY THE PRINICPAL INVESTIGATOR.** | |

**Format of SAE narrative**

1. **Subject details**
   1. Subject initials & other relevant identifier*
   2. Gender
   3. Age and/or date of birth
   4. Weight
   5. Height
2. **Suspected Drug(s)**
   1. Generic name of the drug*
   2. Indication(s) for which suspect drug was prescribed or tested
   3. Dosage form and strength
   4. Daily dose and regimen (specify units - e.g., mg, ml, mg/kg)
   5. Route of administration
   6. Starting date and time of day
   7. Stopping date and time, or duration of treatment
3. **Other Treatment(s)**

Provide the same information for concomitant drugs (including non-prescription/OTC drugs) and non-drug therapies, as for the suspected drug(s).

1. **Details of Serious Adverse Event (s)**

Full description of reaction (s) including body site and severity, as well as the criterion (or criteria) for regarding the report as serious has to be provided. In addition to a description of the reported signs and symptoms, whenever possible, describe a specific diagnosis for the reaction.* Causality assessment by the investigator.

- 1. Start date (and time) of onset of event
  2. Stop date (and time) or duration of event
  3. Dechallenge and rechallenge information
  4. Setting (e.g., hospital, out-patient clinic, home, nursing home)
  5. Results of specific tests and/or treatment that may have been conducted

1. **Outcome**

Information on recovery and any sequelae; for a fatal outcome, cause of death and a comment on its relationship to the suspected reaction; any post-mortem findings

Other information: anything relevant to facilitate assessment of the case, such as medical history including allergy, drug or alcohol abuse; family history; findings from special investigations etc.

**Details of compensations provided for injury or death. In case no compensation has been paid, reason for the same should be submitted. It is pertinent to mention that in case of study related injury or death, complete medical care as well as compensation for the injury or death should be provided.**

1. **Details about the Investigator***
   1. Name, Address & Telephone number
   2. Profession (specialty)
   3. Date of reporting the event to Licensing Authority
   4. Date of reporting the event to Ethics Committee overseeing the site:
   5. Signature of the Investigator

**Note:** Information marked * must be provided
